# The use of LNG-IUS-19.5 mg in daily gynecological routine practice in Germany: data from the Kyleena™ Satisfaction Study (KYSS)

**DOI:** 10.1007/s00404-024-07421-5

**Published:** 2024-02-29

**Authors:** Thomas Römer, Ann-Kathrin Frenz, Susanne Dietrich-Ott, Anja Fiedler

**Affiliations:** 1 Obstetrics and Gynecology Department, Academic Hospital Weyertal, University of Cologne, Cologne, Germany; 2 Medical Affairs, Bayer AG, Berlin, Germany; 3 Jenapharm GmbH & Co. KG, Jena, Germany; 4 Medical Practice of Obstetrics and Gynecology, Gera/Jena, Germany

**Keywords:** Contraception, Low-dose intrauterine system, Levonorgestrel-releasing intrauterine system, Long-acting reversible contraceptive, Contraceptive safety, Satisfaction

## Abstract

**Purpose:**

The Kyleena™ Satisfaction Study (KYSS) provided the first data on 19.5 mg levonorgestrel-releasing intrauterine system (LNG-IUS-19.5 mg) use in routine clinical practice. Here we report results from the German participants in KYSS.

**Methods:**

This prospective, observational, single-arm cohort study recruited women who independently chose to use LNG-IUS-19.5 mg during routine counseling in Germany. Overall satisfaction and bleeding profile satisfaction, continuation rates, and safety profile were evaluated at 12 months or premature end of observation (EoO).

**Results:**

In the German study population, LNG-IUS-19.5 mg placement was attempted in 508 women and successful in 506 women. Mean age was 32.3 years, and 60.0% (n = 305/508) were parous. Placement was considered easy and associated with no more than mild pain, even in younger and nulliparous participants. Of those with satisfaction data available, 87.6% (n = 388/443) were satisfied with LNG-IUS-19.5 mg at 12 months/EoO. Satisfaction was similar for parous (86.9%, n = 238/274) and nulliparous (88.8%, n = 150/169) women, and was independent of age, prior contraceptive method, or reason for choosing LNG-IUS-19.5 mg. Most participants (73.6%, n = 299/406) were also satisfied with their bleeding profile at 12 months/EoO, independent of parity, age, prior contraceptive method, presence of amenorrhea or dysmenorrhea severity. The 12-month continuation rate was 84.1% (n = 427/508). Most discontinuations were due to loss to follow-up (8.5%, n = 43/508) or treatment-emergent adverse events (TEAEs) (4.7%, n = 24/508). TEAEs were reported in 12.6% (n = 64) of participants, with 9.3% (n = 47) considered to have an LNG-IUS-19.5 mg-related TEAE.

**Conclusion:**

Our real-world findings on LNG-IUS-19.5 mg use in German KYSS participants reflected its suitability for a broad population, including young and nulliparous women.

**Clinical trial registration:**

NCT03182140 (date of registration: June 2017).

**Supplementary Information:**

The online version contains supplementary material available at 10.1007/s00404-024-07421-5.

## What does this study add to the clinical work

**Table:** 

The Kyleena™ Satisfaction Study (KYSS) provided the first real-world data on 19.5mg levonorgestrel-releasing intrauterine system (LNG-IUS-19.5mg) use in routine clinical practice in Germany. These data demonstrated the suitability of LNG-IUS-19.5mg for a broad population, including young and nulliparous women.	

## Introduction

Long-acting reversible contraceptive (LARC) methods, which include subdermal implants and both hormonal and non-hormonal intrauterine contraceptives, provide highly effective, long-term, reversible, behavior-independent birth control [1, 2]. LARCs are associated with high satisfaction and continuation rates as well as improved quality of life [3–9]. LARCs, particularly intrauterine contraceptives, are recommended in various international guidelines [10–13], including a joint guideline of the German, Austrian, and Swiss Societies for Gynecology and Obstetrics [14]. Despite these recommendations, intrauterine contraception accounts for only 14% of all contraceptive users worldwide [15].

LARCs are still considerably underused in Germany compared with user-dependent methods such as pills and barrier methods [16, 17]. Recent German studies have demonstrated a trend towards decreasing use of the contraceptive pill, as women increasingly desire a contraceptive method without hormones or with the lowest possible hormone dose [16, 17]. Meanwhile, the newest data from the German Federal Center for Health Education showed that in 2023 the condom was the most common contraceptive method, followed by the pill [18]. As the proportion of women using user-dependent methods of lower efficacy increases, a similar increase has been reflected in the number of abortions in Germany [19].

Barriers and misperceptions persist among both healthcare practitioners (HCPs) and patients, which limit the wider application of levonorgestrel-releasing intrauterine system (LNG-IUS). These include the suitability of LNG-IUS for nulliparous women, pain and difficulty during placement, and concerns about adverse events (AEs) such as pelvic inflammatory disease, expulsion, or uterine perforation [20–26]. These fears are rarely evidence-based, and favorable safety and efficacy profiles have been demonstrated in a broad population, including young and nulliparous women [7, 8, 27–29].

LNG-IUS-19.5 mg (Kyleena™) is indicated for contraception for up to 5 years [30]. It was developed with a smaller T-body (frame size) as well as narrower hormone reservoir and insertion tube than LNG-IUS-52 mg [30], aiming to provide an additional IUS choice for women with a narrower cervical canal and/or smaller uterine cavity, including nulliparous women. LNG-IUS-19.5 mg placement is easy and associated with minimal menstruation-like pain during clinical studies [30, 31]. LNG-IUS-19.5 mg also provides a lower level of levonorgestrel than LNG-IUS-52 mg while maintaining a favorable bleeding profile [5, 6, 30, 32].

The Kyleena™ Satisfaction Study (KYSS) is a multinational, observational study (NCT03182140) providing the first real-world evidence of satisfaction with LNG-IUS-19.5 mg in routine clinical practice [31, 33]. Previously published data from the multinational overall cohort from KYSS showed high levels of satisfaction with LNG-IUS-19.5 mg, high continuation rates, and placement that was generally considered easy with little to no pain; however, these results varied notably between country-specific cohorts [31, 33]. To further examine this variability, here we reported a subgroup analysis and relevant differences from the German participants in KYSS.

## Methods

KYSS was a prospective, multinational, single-arm, observational study with a 1-year follow-up conducted in Belgium, Canada, Germany, Mexico, Norway, Sweden, Spain, and the USA from 2017 to 2018. KYSS assessed LNG-IUS-19.5 mg overall user satisfaction, bleeding profile satisfaction, continuation rates, and safety profile in routine clinical practice. Here we focused on the results from the German participants.

The methodology of this study has been described in detail in previously published analyses [31, 33]. During routine counseling with their HCPs, women who independently chose to use LNG-IUS-19.5 mg were subsequently informed about the study and invited to participate. Exclusion criteria included contraindications for LNG-IUS, mental incapacity to consent, and participation in other clinical trials with interventions outside routine clinical practice.

The primary endpoint for this study was overall user satisfaction rate with LNG-IUS-19.5 mg at the end of observation (EoO) – i.e., 12 months after placement or at premature discontinuation. Secondary endpoint analyses included satisfaction with LNG-IUS-19.5 mg at 12 months/EoO stratified by parity and age. Other endpoints included satisfaction stratified by contraceptive method used in the prior 3 months and motivation for initiating LNG-IUS-19.5 mg use, bleeding profile satisfaction at 12 months/EoO, as well as the ease and pain at placement measures. Safety data including AEs and reasons for early discontinuation were collected. Data on AEs were reported spontaneously by the participants or their HCPs.

Satisfaction ratings were based on the 5-item Likert scale [34]: “very satisfied”, “somewhat satisfied”, “neither satisfied nor dissatisfied”, “dissatisfied”, or “very dissatisfied”. For ratings of ease and pain at LNG-IUS-19.5 mg placement, women reported the levels of pain as “none”, “mild”, “moderate”, or “severe”; whereas the ease of placement was assessed by clinicians using the categories “easy”, “slightly difficult”, or “very difficult”. Participants were asked to assess their menstrual cramps or pain since LNG-IUS-19.5 mg placement at the 4–12-week follow-up visit and for the 3 months prior to EoO. Dysmenorrhea was rated as “none”, “mild”, “moderate”, or “severe”. Similarly, participants were asked whether they had experienced bleeding since LNG-IUS-19.5 mg placement at the 4–12-week follow-up visit and during the 3 months prior to EoO.

Statistical analyses were performed using SAS® software, version 9.4 (Statistical Analysis Systems Institute, Cary, NC, USA) and generic macros [35].

## Results

### Baseline demographics and study population

Overall, 508 participants in Germany had an LNG-IUS-19.5 mg placement attempt (Fig. S1). These participants comprised the safety analysis set (SAF). Mean age was 32.3 years, with 27.8% (n = 141) of the participants under 26 years old, and over half (60.0%, n = 305) were parous (Table 1). Birth control was used by 74.4% (n = 378) of participants in the 3 months prior to enrollment, with the predominant methods being oral contraceptives (30.1%, n = 153), followed by IUS (18.7%, n = 95), and barrier methods (17.7%, n = 90) (Fig. **S2**).

**Table 1:** Baseline demographics of the German population (safety analysis set)^a^

Characteristic	German participants (n = 508)
*Age, years, mean* ± *SD* *Age, years, n (%)* ≤ 17 18–25 26–35 > 35	32.3 ± 8.8 **5 (1.0)** **136 (26.8)** 182 (35.8) 185 (36.4)
*Parity, n (%)* Parous Nulliparous	305 (60.0) **203 (40.0)**
*BMI, kg/m*^*2*^*, mean* ± *SD*	24.5 ± 4.7
*Previous contraception during last 3 months, n (%)* Yes No	378 (74.4) 130 (25.6)

^a^Note that data from categories of younger and nulliparous women are highlighted in bold as these are of particular interest in this publication

*BMI* body mass index, *SD* standard deviation

The most common reasons for selecting LNG-IUS-19.5 mg were desires to avoid the need for a contraceptive routine (34.3%, n = 174), for high contraceptive reliability (31.3%, n = 159), and for low hormone dose (31.1%, n = 158) (Fig. **S3**). Also noted as important reasons were the expectation of shorter, lighter, and less frequent bleeding episodes (19.1%, n = 97), and the mainly local rather than systemic effects of LNG (14.2%, n = 72).

### LNG-IUS-19.5 mg placement experience

LNG-IUS-19.5 mg placement was successful in 506 of the 508 participants (99.6%); these participants comprised the full analysis set (FAS). Investigators rated the majority (90.2%, n = 458) of placements as “easy” (Fig. **S4**). Ease of placement was similar regardless of the woman's age or parity. Congruently, most women (81.9%, n = 416) stated that they had felt no or “mild” pain during placement. In total, 14.2% (n = 72) of participants rated the placement pain as “moderate”, and only 3.9% (n = 20) felt “severe” pain. Parous women more frequently rated their pain as “none” or “mild” (89.8%, n = 274/305) in comparison with nulliparous participants (70.0%, n = 142/203). This was also true for older (aged > 25 years) in comparison with younger (aged ≤ 25 years) participants: 84.7% (n = 311/367) and 74.5% (n = 105/141), respectively, rated their pain as “none” or “mild”.

Concomitant medication or additional measures such as cervical dilation for insertion were not required in most German women (59.1%, n = 300) (Fig. **S4**). Systemic medication (e.g., non-steroidal anti-inflammatory drugs or prostaglandin analogs) was used in 23.0% (n = 117) of placements; local medication (e.g., local anesthesia gel or fluid) was used in 11.8% (n = 60); and cervical dilation was used in 3.3% (n = 17). Nulliparous women were more likely to require additional interventions than parous women: 48.8% (n = 99/203) and 35.7% (n = 109/305), respectively. This was also true for younger (≤ 25 years) compared with older (> 25 years) women: 49.6% (n = 70/141) and 37.6% (n = 138/367), respectively, required additional measures.

### Satisfaction with LNG-IUS-19.5 mg

**Fig. 1:**
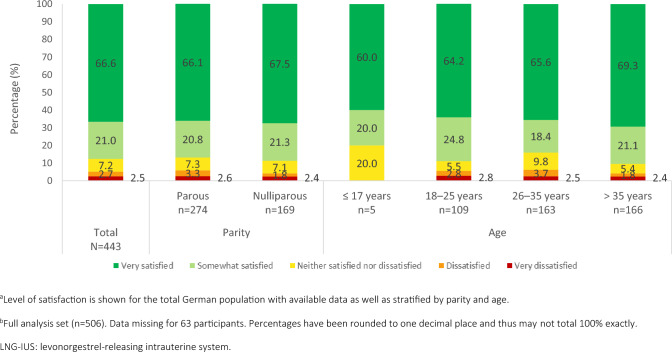
Satisfaction of German participants with LNG-IUS-19.5 mg at 12 months/end of observation (full analysis set)^a,b^

Most participants at 12 months/EoO were satisfied with LNG-IUS-19.5 mg; 87.6% (n = 388) of the 443 FAS participants with available satisfaction data reported that they were satisfied with the device (Fig. 1). The majority of women (66.6%, n = 295) reported being “very satisfied” with LNG-IUS-19.5 mg, with a very low proportion (2.5%, n = 11) reporting being “very dissatisfied” with LNG-IUS-19.5 mg at 12 months/EoO. However, it should be noted that no satisfaction data were available for 12.5% of women (63 of 506 FAS participants), mainly due to loss to follow-up.

Most German women reported satisfaction at 12 months/EoO (Fig. 1), irrespective of parity; 86.9% (n = 238/274) of parous women and 88.8% (n = 150/169) of nulliparous women reported satisfaction. Similarly, most participants reported satisfaction regardless of age, ranging from 80.0% (n = 4/5) of women aged ≤ 17 years to 90.4% (n = 150/166) of those aged > 35 years.

**Fig. 2:**
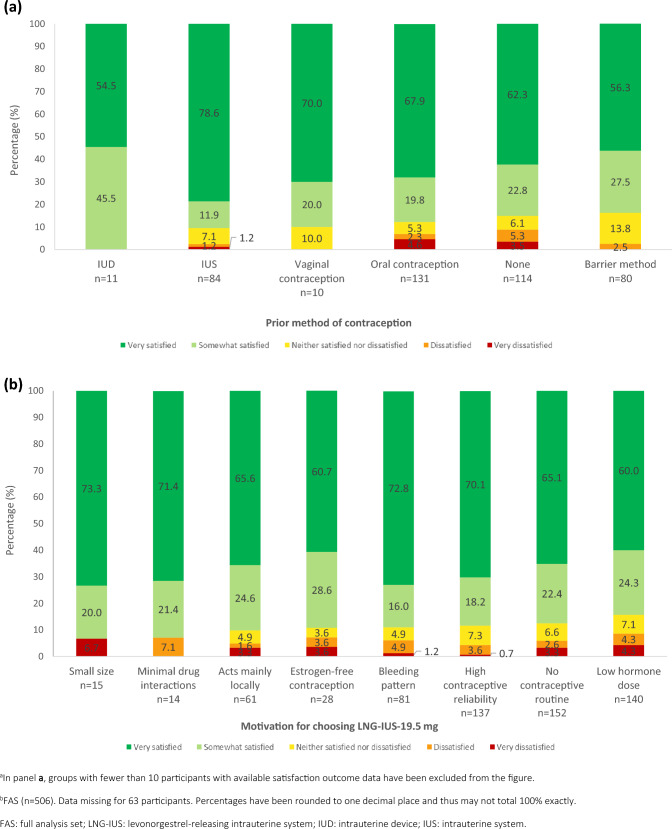
Satisfaction of German participants with LNG-IUS-19.5 mg at 12 months/end of observation (FAS), by subgroups. **a** Level of satisfaction stratified by previous contraceptive method^a,b^ ; **b** Level of satisfaction stratified by motivation for choosing LNG-IUS-19.5mg^b^

When LNG-IUS-19.5 mg satisfaction was compared with all previous methods of contraception, the vast majority of participants were satisfied irrespective of the method used (Fig. 2A). Across all prior contraceptive methods, at least 83.8% reported satisfaction, with satisfaction being highest in previous IUS (90.5%, n = 76/84), and IUD (100%, n = 11/11) users.

Although various reasons for selecting LNG-IUS-19.5 mg were given, satisfaction was similar when stratified by these motivations for choice (Fig. 2B). Satisfaction ranged from 84.3% (n = 118/140) for those who chose LNG-IUS-19.5 mg for its low hormone dose to 93.3% (n = 14/15) for those who chose LNG-IUS-19.5 mg for its small size.

### Bleeding profile satisfaction

**Fig. 3:**
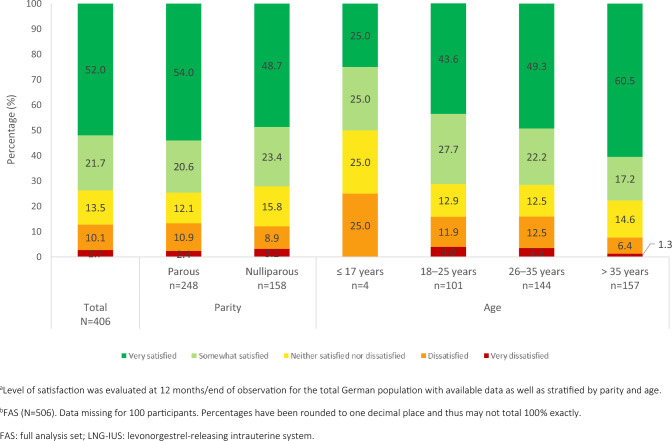
Satisfaction of German participants with their bleeding profile during LNG-IUS-19.5 mg use (FAS)^a,b^

At 12 months/EoO, 73.6% (n = 299/406) of women with satisfaction outcome data available reported being satisfied with their bleeding profile (Fig. 3). Bleeding profile satisfaction was independent of parity, with 74.6% (n = 185/248) of parous women and 72.1% (n = 114/158) of nulliparous women reporting being “very satisfied” or “somewhat satisfied”. When stratified by age, bleeding profile satisfaction ranged from 50.0% (n = 2/4) for those aged ≤ 17 years to 77.7% (n = 122/157) for those aged > 35 years.

Bleeding profile satisfaction differed when stratified by prior contraceptive method (Table S1). Previous users of an IUS or IUD had the highest satisfaction rates: 86.7% (n = 65/75) and 81.8% (n = 9/11), respectively, reported being “very satisfied” or “somewhat satisfied”. Those who had not been using any contraceptive method in the past 3 months and those who had been using barrier methods reported the lowest satisfaction rates, although the majority were still satisfied: 65.7% (n = 67/102) and 64.1% (n = 50/78) reported satisfaction, respectively.

Analysis of bleeding profile satisfaction was additionally stratified by participants’ reports of whether they had experienced bleeding since LNG-IUS-19.5 mg placement (Table S1). Rates of satisfaction were similar between those with amenorrhea and those without: 74.2% (n = 89/120) and 73.5% (n = 200/272), respectively, reported being “very satisfied” or “somewhat satisfied”. Bleeding profile satisfaction was also stratified by dysmenorrhea severity (Table S1). The proportion of participants who were “very satisfied” decreased with increasing dysmenorrhea severity, although most participants reported satisfaction regardless of dysmenorrhea severity. The proportion of women being “very satisfied” ranged from 42.4% (n = 14/33) for those with severe dysmenorrhea to 57.8% (n = 93/161) for those with no dysmenorrhea.

### Continuation with LNG-IUS-19.5 mg

In total, 84.1% (n = 427/508) of participants chose to continue with LNG-IUS-19.5 mg at 12 months (Table 2). Of the 15.9% (n = 81/508) who did not complete the planned final study visit at 12 months, 8.5% (n = 43) were lost to follow-up and may or may not have continued with the device. Only four women (0.8%) discontinued for the primary reason of being dissatisfied with LNG-IUS-19.5 mg.

**Table 2:** Continuation with LNG-IUS-19.5 mg at 12 months/end of observation in the German population (safety analysis set)^a^

Outcome, n (%)	German participants (n = 508)
*LNG-IUS-19.5 mg still in use at planned EoO*	427 (84.1)
*LNG-IUS-19.5 mg discontinued before planned EoO*	81 (15.9)
*Primary reason for EoO/discontinuation:* Lost to follow-up Unsuccessful LNG-IUS-19.5 mg placement Expulsion of LNG-IUS-19.5 mg Removal of LNG-IUS-19.5 mg Adverse event Dissatisfaction with LNG-IUS-19.5 mg Pregnancy Wish for pregnancy Switch contraceptive method Uterine perforation	43 (8.5) 2 (0.4) **3 (0.6)** 33 (6.5) 20 (3.9) 4 (0.8) **2 (0.4)** 4 (0.8) 3 (0.6) **0**

^a^Note that events of particular interest in this publication are highlighted in bold

*EoO* end of observation, *LNG-IUS* levonorgestrel-releasing intrauterine system

### Adverse events with LNG-IUS-19.5 mg

Treatment emergent adverse events (TEAEs) were reported in 12.6% (n = 64/508) of participants. Discontinuations due to TEAEs were uncommon: 4.7% (n = 24/508) of participants discontinued treatment due to TEAEs (Table 3). Few discontinuations were observed due to bleeding-related TEAEs (1.2%, n = 6). There were three expulsions (0.6%), two pregnancies (0.4%), two infections (0.4%), and no uterine perforations (Table 2). Of the two pregnancies, one was ectopic and required remedial drug therapy; the other was intrauterine and resulted in spontaneous abortion.

**Table 3:** Adverse events and discontinuation rates with LNG-IUS-19.5 mg in the German population (safety analysis set)

Outcome, n (%)	German participants (n = 508)
*Any TEAE*	64 (12.6)
*Any LNG-IUS-19.5 mg-related TEAE*	47 (9.3)
*Any serious TEAE*	6 (1.2)
*Any serious LNG-IUS-19.5 mg-related TEAE*	3 (0.6)
*Discontinuation due to TEAE*	24 (4.7)
*Discontinuation due to serious TEAE*	3 (0.6)
*Discontinuation due to bleeding-related TEAE*	6 (1.2)

*LNG-IUS* levonorgestrel-releasing intrauterine system, *TEAE* treatment-emergent adverse event

In total, 9.3% (n = 47) were considered to have an LNG-IUS-19.5 mg-related TEAE (Table 3), with disorders of the reproductive system and the mammary gland being the most common (reported in 5.1%, n = 26) (Table **S2**). This class of AEs included metrorrhagia, ovarian cysts, and vaginal bleeding (each reported in 0.8%, n = 4), as well as dysmenorrhea (0.6%, n = 3). Gastrointestinal disorders (3.3%, n = 17) such as lower abdominal pain (3.1%, n = 16), skin disorders (1.0%, n = 5) such as acne (0.4%, n = 2), and psychiatric disorders (0.8%, n = 4) such as depression (0.2%, n = 1) were also among the reported LNG-IUS-19.5 mg-related TEAEs.

## Discussion

KYSS provided the first real-world evidence on the use of LNG-IUS-19.5 mg in an international population. The results reported here for the German participants demonstrated high satisfaction rates with LNG-IUS-19.5 mg regardless of age, parity, previous contraceptive method, or motivation for initiating LNG-IUS-19.5 mg use. In addition, these data showed that most participants were satisfied with their bleeding profile during LNG-IUS-19.5 mg use, even when stratified by age, parity, prior contraceptive method, presence of amenorrhea, and severity of dysmenorrhea.

As Germany represented the largest country-specific KYSS population with 508 of the total 1129 participants, these data reflected the most important conclusions of the multinational KYSS population [33]. These data also further supported the high satisfaction and continuation rates as well as the favorable safety profile demonstrated with LNG-IUS-19.5 mg in prior clinical trials [6, 7, 32]. Although real-world evidence on satisfaction with LNG-IUS is still limited, satisfaction with an LNG-IUS (not specific to LNG-IUS-19.5 mg) has previously been demonstrated in everyday practice in Germany, with prior studies showing high satisfaction with LNG-IUS that compared favorably with other contraceptive methods. Consistent with this, our results provided the first real-world evidence specific to LNG-IUS-19.5 mg to support these previous studies.

Concerns about nulliparity and difficult or painful placement are among the main barriers deterring women and clinicians from considering intrauterine contraception [20–26]. Our data demonstrated ease of placement in routine clinical practice and showed that there was usually minimal pain during the placement procedure. Importantly, this was independent of age and parity. Although nulliparous participants were more likely to report slightly higher levels of pain during placement, the majority of young and/or nulliparous participants reported no or “mild” pain. Moreover, most participants did not require any pain-managing interventions. This was again largely independent of age or parity, although additional measures for managing pain were more likely to be required by younger or nulliparous participants. These findings would be useful for contraceptive counseling on IUS placement procedures; HCPs can be assured that placements are generally easy and associated with no more than mild pain.

A previous survey has demonstrated a common belief among HCPs that intrauterine contraceptive use in nulliparous women is associated with a higher risk for pelvic inflammatory disease, infertility, and uterine perforation [21]. Here, we showed that the German participants in KYSS had a low rate of discontinuations and TEAEs; in particular, the incidences of pelvic inflammatory disease, expulsions, and unsuccessful placements compared very favorably with published safety data from clinical trials [6, 7, 32], which is often rare for real-world evidence in routine clinical settings. We observed no perforations within both the German subset and the entire KYSS population; this was also consistent with the real-world large-scale European Active Surveillance Study for Intrauterine Devices (EURAS-IUD), which reported a perforation rate of 2.1 per 1000 LNG-IUS insertions [36]. Additionally, rates of depression and mood disorders were low in KYSS. These results were particularly positive given that psychiatric-related AEs with use of hormonal contraceptives are another common area of concern for HCPs [18, 37]. Our data thus provided further evidence to show that LNG-IUS-19.5 mg is well tolerated with a favorable safety profile and should help to alleviate misconceptions among HCPs regarding intrauterine contraception.

Furthermore, satisfaction with the bleeding profile during LNG-IUS-19.5 mg use was high, regardless of age, parity, the presence of amenorrhea, or the severity of any dysmenorrhea. There were a low number of bleeding-related TEAEs considered to be due to LNG-IUS-19.5 mg use, and few participants discontinued as a result of bleeding-related AEs. This was an important finding because concerns about bleeding profile changes have been identified as a barrier to IUS use [38, 39].

We demonstrated that satisfaction with LNG-IUS-19.5 mg use was independent from the reasons of choice. This was observed in the German subset as well as the entire multinational KYSS population; however, there were relevant differences between these two populations [33]. Of note, the desires for high contraceptive reliability (31.4 vs. 27.6%), low hormone dose (31.2 vs. 26.6%), and a mainly local contraceptive effect (14.2 vs. 10.0%) were expressed more frequently by German participants com-pared with the total population. This provided insights into the trends of the contraceptive landscape in Germany and further evidence that indicates changes in patient attitudes and increasing hormone skepticism [16, 17, 40]. Importantly, such findings would be useful for HCPs since there is a considerable body of evidence supporting the influence of provider counseling on women’s contraceptive method choices [41–45] and how shared decision-making promotes patient satisfaction [46]; these, in turn, are inversely associated with the discontinuation of contraceptive methods [4, 47–49]. Understanding the factors influencing a woman’s contraceptive choice will allow providers to dispel misconceptions, counsel more effectively, and help women select the contraceptive method most suitable for their needs.

Recent trends from Germany showed an increasing proportion of women using condoms as their primary contraceptive method [18] (and a concomitant increase in abortions [19]). The drivers for these trends are likely multifaceted but the rise in hormone skepticism may be partially responsible [16, 17, 40]. Indeed, our study found that low hormone dose was a key priority when selecting a contraceptive method; however, high contraceptive reliability was also an important factor. LNG-IUS-19.5 mg could, therefore, be a valuable option for German women as its low hormone dose does not prevent ovulation, so women can maintain their natural cycles while still benefitting from highly effective contraception [30].

Limitations of this study included the unavailability of satisfaction data for some participants due to loss- to-follow-up, which may impact the satisfaction rates. The study population was also relatively homogeneous in terms of body mass index and ethnicity; therefore, results may not be generalizable to other populations. However, KYSS was not designed to investigate the impact of race, ethnicity, education level, or socioeconomic status on satisfaction rates.

## Conclusion

We reported high overall satisfaction with LNG-IUS-19.5 mg for the German participants in KYSS, independent of age, parity, prior contraceptive method, or reasons of choice. Bleeding profile satisfaction during LNG-IUS-19.5 mg use was also high, regardless of the presence of amenorrhea and the severity of any dysmenorrhea. LNG-IUS-19.5 mg placement was generally easy and mostly associated with mild to no pain, even in younger and nulliparous participants. A favorable safety profile and high continuation rates were also observed. Together, these real-world data on LNG-IUS-19.5 mg use in German participants in routine clinical practice underscored its suitability for a broad population, including young and nulliparous women.

### Supplementary Information

Below is the link to the electronic supplementary material.

Supplementary file1 (DOCX 84 kb)

## Data Availability

Availability of the data underlying this publication is determined according to Bayer’s commitment to the EFPIA/PhRMA “Principles for responsible clinical trial data sharing”. This pertains to scope, time point and process of data access. As such, Bayer commits to sharing upon request from qualified scientific and medical researchers patient-level clinical trial data, study-level clinical trial data, and protocols from clinical trials in patients for medicines and indications approved in the United States (US) and European Union (EU) as necessary for conducting legitimate research. This applies to data on new medicines and indications that have been approved by the EU and US regulatory agencies on or after January 01, 2014. Interested researchers can use www.clinicalstudydatarequest.com to request access to anonymised patient-level data and supporting documents from clinical studies to conduct further research that can help advance medical science or improve patient care. Information on the Bayer criteria for listing studies and other relevant information is provided in the Study sponsors section of the portal (www.clinicalstudydatarequest.com/Study-Sponsors.aspx). Data access will be granted to anonymised patient-level data, protocols and clinical study reports after approval by an independent scientific review panel. Bayer is not involved in the decisions made by the independent review panel. Bayer will take all necessary measures to ensure that patient privacy is safeguarded.

## References

[1] Trussell JC (2011). Contraceptive failure in the United States. Contraception.

[2] Winner B, Peipert JF, Zhao Q, Buckel C, Madden T, Allsworth JE, Secura GM (2012). Effectiveness of long-acting reversible contraception. N Engl J Med.

[3] Römer T, Linsberger D (2009). User satisfaction with a levonorgestrel-releasing intrauterine system (LNG-IUS): data from an international survey. Eur J Contracept Reprod Health Care.

[4] Peipert JF, Zhao Q, Allsworth JE, Petrosky E, Madden T, Eisenberg D, Secura G (2011). Continuation and satisfaction of reversible contraception. Obstet Gynecol.

[5] Gemzell-Danielsson K, Cho S, Inki P, Mansour D, Reid R, Bahamondes L (2012). Use of contraceptive methods and contraceptive recommendations among health care providers actively involved in contraceptive counseling – results of an international survey in 10 countries. Contraception.

[6] Nelson A, Apter D, Hauck B, Schmelter T, Rybowski S, Rosen K, Gemzell-Danielsson K (2013). Two low-dose levonorgestrel intrauterine contraceptive systems. Obstet Gynecol.

[7] Gemzell-Danielsson K, Apter D, Hauck B, Schmelter T, Rybowski S, Rosen K, Nelson A (2015). The effect of age, parity and body mass index on the efficacy, safety, placement and user satisfaction associated with two low-dose levonorgestrel intrauterine contraceptive systems: subgroup analyses of data from a Phase III trial. PLoS ONE.

[8] Merki-Feld GS, Caetano C, Porz TC, Bitzer J (2018). Are there unmet needs in contraceptive counselling and choice? Findings of the European TANCO Study. Eur J Contracept Reprod Health Care.

[9] Faustmann T, Crocker J, Moeller C, Engler Y, Caetano C, Buhling KJ (2019). How do women and health care professionals view hormonal long-acting reversible contraception? Results from an international survey. Eur J Contracept Reprod Health Care.

[10] National Institute for Health and Care Excellence (NICE) (2005) Clinical guideline CG30: long-acting reversible contraception. [Published: 26 October 2005; last updated: 02 July 2019]. https://www.nice.org.uk/guidance/cg30. Accessed 15 Nov 2023 31999411

[11] Curtis KM, Tepper NK, Jatlaoui TC, Berry-Bibee E, Horton LG, Zapata LB, Simmons KB, Pagano HP, Jamieson DJ, Whiteman MK (2016). U.S. medical eligibility criteria for contraceptive use, 2016. MMWR Morb Mortal Wkly Rep.

[12] American College of Obstetricians and Gynecologists (ACOG) (2015). ACOG Committee Opinion No. 642: Increasing Access to Contraceptive Implants and Intrauterine Devices to Reduce Unintended Pregnancy. Obstet Gynecol.

[13] Black A, Guilbert E, Costescu D, Dunn S, Fisher W, Kives S, Mirosh M, Norman W, Pymar H, Reid R, Roy G, Varto H, Waddington A, Wagner MS, Whelan AM, Mansouri S (2016). Canadian contraception consensus (Part 3 of 4): chapter 7—intrauterine contraception. J Obstet Gynaecol Can.

[14] Franik S, Bauersachs R, Beyer-Westendorf J, Buchholz T, Bühling K, Diener HC, Erath A, Fischer R, Förderreuther S, Franz HBG, Hach-Wunderle V, Hadji P, Harlfinger W, Jaursch-Hancke C, König K, Krämer G, Naumann G, Neulen J, Oppelt PG, Pliefke J, Rimbach S, Rott H, Schroll E, Schumann C, Segerer S, Seyler H, Tempfer C, Thonke I, Toth B, Wildt L, Zotz R, Stute P, Kiesel L (2021). Hormonal contraception. guideline of the DGGG, OEGGG and SGGG (S3 Level, AWMF Registry Number 015/015, January 2020). Geburtsh Frauenheilkd.

[15] United Nations (UN) (2019). Contraceptive use by method 2019.

[16] Oppelt PG, Bitzer J (2021). Aktuelle Trends im Verhütungsverhalten der Frauen in Deutschland und Relevanz einer bedarfsgerechten Verhütungsberatung anhand der Daten von TANCO und CoCo.

[17] Bitzer J, Oppelt PG, Deten A (2021). Evaluation of a patient-centred, needs-based approach to support shared decision making in contraceptive counselling: the COCO study. Eur J Contracept Reprod Health Care.

[18] German Federal Center for Health Education (2023) BZgA-Studie: Verhütungsverhalten Erwachsener 2023 Repräsentative BZgA-Wiederholungsbefragung (in German). https://www.bzga.de/fileadmin/user_upload/PDF/pressemitteilungen/daten_und_fakten/Infoblatt_BZgA-Studiendaten_Verhütungsverhalten_2023.pdf. Accessed Dec 2023

[19] German Federal Statistical Office (2023) Anzahl der Schwangerschaftsabbrüche in Deutschland nach Alter und Quote (in German). https://www.destatis.de/DE/Themen/Gesellschaft-Umwelt/Gesundheit/Schwangerschaftsabbrueche/Tabellen/01-schwangerschaftsabbr-alter-quote-10tsd-je-altersgruppe_zvab2012.html. Accessed Dec 2023

[20] Madden T, Allsworth JE, Hladky KJ, Secura GM, Peipert JF (2010). Intrauterine contraception in Saint Louis: a survey of obstetrician and gynecologists' knowledge and attitudes. Contraception.

[21] Buhling KJ, Hauck B, Dermout S, Ardaens K, Marions L (2014). Understanding the barriers and myths limiting the use of intrauterine contraception in nulliparous women: results of a survey of European/Canadian healthcare providers. Eur J Obstet Gynecol Reprod Biol.

[22] Secura GM, Allsworth JE, Madden T, Mullersman JL, Peipert JF (2010). The Contraceptive CHOICE Project: reducing barriers to long-acting reversible contraception. Am J Obstet Gynecol.

[23] Hladky KJ, Allsworth JE, Madden T, Secura GM, Peipert JF (2011). Women's knowledge about intrauterine contraception. Obstet Gynecol.

[24] Potter J, Rubin SE, Sherman P (2014). Fear of intrauterine contraception among adolescents in New York City. Contraception.

[25] Brima N, Akintomide H, Iguyovwe V, Mann S (2015). A comparison of the expected and actual pain experienced by women during insertion of an intrauterine contraceptive device. Open Access J Contracept.

[26] Zapata LB, Morgan IA, Curtis KM, Folger SG, Whiteman MK (2019). Changes in US health care provider attitudes related to contraceptive safety before and after the release of National Guidance. Contraception.

[27] Aoun J, Dines VA, Stovall DW, Mete M, Nelson CB, Gomez-Lobo V (2014). Effects of age, parity, and device type on complications and discontinuation of intrauterine devices. Obstet Gynecol.

[28] Eisenberg DL, Schreiber CA, Turok DK, Teal SB, Westhoff CL, Creinin MD, Investigators ACCESSIUS (2015). Three-year efficacy and safety of a new 52-mg levonorgestrel-releasing intrauterine system. Contraception.

[29] Lohr PA, Lyus R, Prager S (2017). Clinical Guidelines: Use of intrauterine devices in nulliparous women. Contraception.

[30] Gemzell-Danielsson K, Apter D, Dermout S, Faustmann T, Rosen K, Schmelter T, Merz M, Nelson A (2017). Evaluation of a new, low-dose levonorgestrel intrauterine contraceptive system over 5 years of use. Eur J Obstet Gynecol Reprod Biol.

[31] Beckert V, Aqua K, Bechtel C, Cornago S, Kallner HK, Schulze A, Parashar P, Waddington A, Donders G (2020). Insertion experience of women and health care professionals in the Kyleena® Satisfaction Study. Eur J Contracept Reprod Health Care.

[32] Gemzell-Danielsson K, Schellschmidt I, Apter D (2012). A randomized, phase II study describing the efficacy, bleeding profile, and safety of two low-dose levonorgestrel-releasing intrauterine contraceptive systems and Mirena. Fertil Steril.

[33] Stovall DW, Aqua K, Römer T, Donders G, Sørdal T, Hauck B, Llata ES, Kallner HK, Salomon J, Zvolanek M, Frenz AK, Böhnke T, Bauerfeind A (2021). Satisfaction and continuation with LNG-IUS 12: findings from the real-world Kyleena® Satisfaction Study. Eur J Contracept Reprod Health Care.

[34] Likert RA (1932). A technique for the measurement of attitudes. Arch Psychol.

[35] Liu Y, Nickleach DC, Zhang C, Switchenko JM, Kowalski J (2018). Carrying out streamlined routine data analyses with reports for observational studies: introduction to a series of generic SAS ® macros. F1000Res.

[36] Barnett C, Moehner S, Do Minh T, Heinemann K (2017). Perforation risk and intra-uterine devices: results of the EURAS-IUD 5-year extension study. Eur J Contracept Reprod Health Care.

[37] Ludwig M, Römer T, Neulen J (2020). Depressionen unter der Hormonspirale-Anmerkungen aus gynäkologischer Sicht. Frauenarzt.

[38] Costescu D, Chawla R, Hughes R, Teal S, Merz M (2022). Discontinuation rates of intrauterine contraception due to unfavourable bleeding: a systematic review. BMC Womens Health.

[39] Beckert V, Ahlers C, Frenz AK, Gerlinger C, Bannemerschult R, Lukkari-Lax E (2019). Bleeding patterns with the 19.5mg LNG-IUS, with special focus on the first year of use: implications for counselling. Eur J Contracept Reprod Health Care.

[40] Bundeszentrale für gesundheitliche Aufklärung (BZgA) (2020) Verhütungsverhalten Erwachsener. Ergebnisse der Repräsentativbefragung 2018. https://publikationen.sexualaufklaerung.de/themen/verhuetung/verhuetungsverhalten-erwachsener-2018/. Accessed 4 Aug 2023

[41] Brown MK, Auerswald C, Eyre SL, Deardorff J, Dehlendorf C (2013). Identifying counseling needs of nulliparous adolescent intrauterine contraceptive users: a qualitative approach. J Adolesc Health.

[42] Marshall C, Kandahari N, Raine-Bennett T (2018). Exploring young women's decisional needs for contraceptive method choice: a qualitative study. Contraception.

[43] Rubin SE, Felsher M, Korich F, Jacobs AM (2016). Urban adolescents' and young adults' decision-making process around selection of intrauterine contraception. J Pediatr Adolesc Gynecol.

[44] Cohen R, Sheeder J, Kane M, Teal SB (2017). Factors associated with contraceptive method choice and initiation in adolescents and young women. J Adolesc Health.

[45] Melo J, Peters M, Teal S, Guiahi M (2015). Adolescent and young women's contraceptive decision-making processes: choosing "the best method for her". J Pediatr Adolesc Gynecol.

[46] Dehlendorf C, Grumbach K, Schmittdiel JA, Steinauer J (2017). Shared decision making in contraceptive counseling. Contraception.

[47] Moreau C, Cleland K, Trussell JC (2007). Contraceptive discontinuation attributed to method dissatisfaction in the United States. Contraception.

[48] Rosenberg MJ, Waugh MS (1998). Oral contraceptive discontinuation: A prospective evaluation of frequency and reasons. A prospective evaluation of frequency and reasons. Am J Obstet Gynecol.

[49] Huber LRB, Hogue CJ, Stein AD (2006). Contraceptive use and discontinuation: findings from the contraceptive history, initiation, and choice study. Am J Obstet Gynecol.

