# Supplementary material for: The use of LNG-IUS-19.5 mg in daily gynecological routine practice in Germany: data from the Kyleena™ Satisfaction Study (KYSS)
Source: Arch Gynecol Obstet. 2024 Feb 29;309(5):2021–30. doi: 10.1007/s00404-024-07421-5 (PMC11018657; doi:10.1007/s00404-024-07421-5)
Supplement: Supplementary file 1 — Supplementary file1 (DOCX 84 kb) [file 404_2024_7421_MOESM1_ESM.docx]

**SUPPLEMENTARY MATERIAL**

**The Use of LNG-IUS-19.5mg in Daily Gynecological Routine Practice in Germany: Data from the Kyleena™ Satisfaction Study (KYSS)**

**Thomas Römer,^1^ Ann-Kathrin Frenz,^2^ Susanne Dietrich-Ott,^3^ Anja Fiedler^4^**

Affiliations: ^1^Obstetrics and Gynecology Department, Academic Hospital Weyertal, University of Cologne, Cologne, Germany; ^2^Medical Affairs, Bayer AG, Berlin, Germany; ^3^Jenapharm GmbH & Co. KG, Jena, Germany; ^4^Medical Practice of Obstetrics and Gynecology, Gera/Jena, Germany

Correspondence to: Thomas Römer, [Thomas.Roemer@evk-koeln.de](mailto:Thomas.Roemer@evk-koeln.de)

**Table S1** Satisfaction of German participants with their bleeding profile during LNG-IUS-19.5mg use at 12 months/end of observation (FAS) by previous contraceptive method, by presence of amenorrhea and by dysmenorrhea severity

| **Satisfaction with bleeding profile, n (%)** | | **German participants, n** | **Very satisfied** | **Somewhat satisfied** | **Neither satisfied nor dissatisfied** | **Dissatisfied** | **Very dissatisfied** |
| --- | --- | --- | --- | --- | --- | --- | --- |
| **Previous contraceptive method^a,b^** | **None** | **102** | 43 (42.2) | 24 (23.5) | 19 (18.6) | 12 (11.8) | 4 (3.9) |
|  | **Oral contraception** | **118** | 64 (54.2) | 27 (22.9) | 14 (11.9) | 9 (7.6) | 4 (3.4) |
|  | **Barrier method** | **78** | 33 (42.3) | 17 (21.8) | 11 (14.1) | 15 (19.2) | 2 (2.6) |
|  | **IUS** | **75** | 51 (68.0) | 14 (18.7) | 6 (8.0) | 3 (4.0) | 1 (1.3) |
|  | **IUD** | **11** | 6 (54.5) | 3 (27.3) | 1 (9.1) | 1 (9.1) | 0 |
|  | **Vaginal contraception** | **10** | 5 (50.0) | 2 (20.0) | 2 (20.0) | 1 (10.0) | 0 |
| **Presence of amenorrhea^b^** | **Yes** | **120** | 64 (53.3) | 25 (20.8) | 14 (11.7) | 13 (10.8) | 4 (3.3) |
|  | **No** | **272** | 140 (51.5) | 60 (22.1) | 39 (14.3) | 26 (9.6) | 7 (2.6) |
| **Dysmenorrhea severity^c^** | **No dysmenorrhea** | **161** | 93 (57.8) | 30 (18.6) | 20 (12.4) | 14 (8.7) | 4 (2.5) |
|  | **Mild** | **111** | 57 (51.4) | 25 (22.5) | 17 (15.3) | 9 (8.1) | 3 (2.7) |
|  | **Moderate** | **88** | 41 (46.6) | 18 (20.5) | 15 (17.0) | 12 (13.6) | 2 (2.3) |
|  | **Severe** | **33** | 14 (42.4) | 11 (33.3) | 3 (9.1) | 4 (12.1) | 1 (3.0) |

FAS: full analysis set; IUD: intrauterine device; IUS: intrauterine system; LNG-IUS: levonorgestrel-releasing intrauterine system.

^a^Groups with fewer than 10 participants with available bleeding satisfaction outcome data have been excluded from the table.

^b^Data missing for 114 participants.

^c^Data missing for 113 participants.

**Table S2** Summary of the LNG-IUS-19.5mg–related TEAEs at 12 months/premature discontinuation for the German population and overall population in KYSS (safety analysis set)^a,b^

| TEAE, n (%) | German participants (n=508) |
| --- | --- |
| *Any LNG-IUS-19.5mg–related TEAE* | **47 (9.3)** |
| *Reproductive system and breast disorders*  Cervical dysplasia  Dysmenorrhea  Genital hemorrhage  Hypomenorrhea  Menorrhagia  Menstrual disorder  Metrorrhagia  Ovarian cyst  Ovarian cyst ruptured  Pelvic pain  Uterine hemorrhage  Vaginal hemorrhage  Vulvovaginal burning sensation | 26 (5.1)  1 (0.2)  **3 (0.6)**  1 (0.2)  1 (0.2)  6 (1.2)  2 (0.4)  4 (0.8)  **4 (0.8)**  1 (0.2)  1 (0.2)  3 (0.6)  4 (0.8)  1 (0.2) |
| *Gastrointestinal disorders*  Abdominal pain  Abdominal pain lower  Abdominal pain upper  Flatulence | 17 (3.3)  1 (0.2)  16 (3.1)  1 (0.2)  1 (0.2) |
| *Skin and subcutaneous tissue disorders*  Acne  Alopecia  Skin disorder | 5 (1.0)  **2 (0.4)**  1 (0.2)  2 (0.4) |
| *Psychiatric disorders*  Depressed mood  Depression  Listless  Panic attack | **4 (0.8)**  **1 (0.2)**  **1 (0.2)**  **1 (0.2)**  **1 (0.2)** |
| *Cardiac disorders*  Cardiovascular disorder | 2 (0.4)  2 (0.4) |
| *Infections*  Endometritis  Salpingo-oophoritis | **2 (0.4)**  **1 (0.2)**  **1 (0.2)** |
| *Nervous system disorders*  Headache  Migraine | **2 (0.4)**  **1 (0.2)**  **1 (0.2)** |
| *Product issues*  Device dislocation | 2 (0.4)  **2 (0.4)** |
| *Ear and labyrinth disorders*  Vertigo | 1 (0.2)  1 (0.2) |
| *Musculoskeletal and connective tissue disorders*  Back pain | 1 (0.2)  1 (0.2) |
| *Vascular disorders*  Hypotension | 1 (0.2)  1 (0.2) |

KYSS: Kyleena^TM^ Satisfaction Study; LNG-IUS: levonorgestrel-releasing intrauterine system; TEAE: treatment-emergent adverse event.

^a^KYSS was conducted in Belgium, Canada, Germany, Mexico, Norway, Sweden, Spain, and the United States.

^b^Note that the adverse events of particular interest in this publication are highlighted in bold.

**Fig. S1** Flow chart of German participants in KYSS


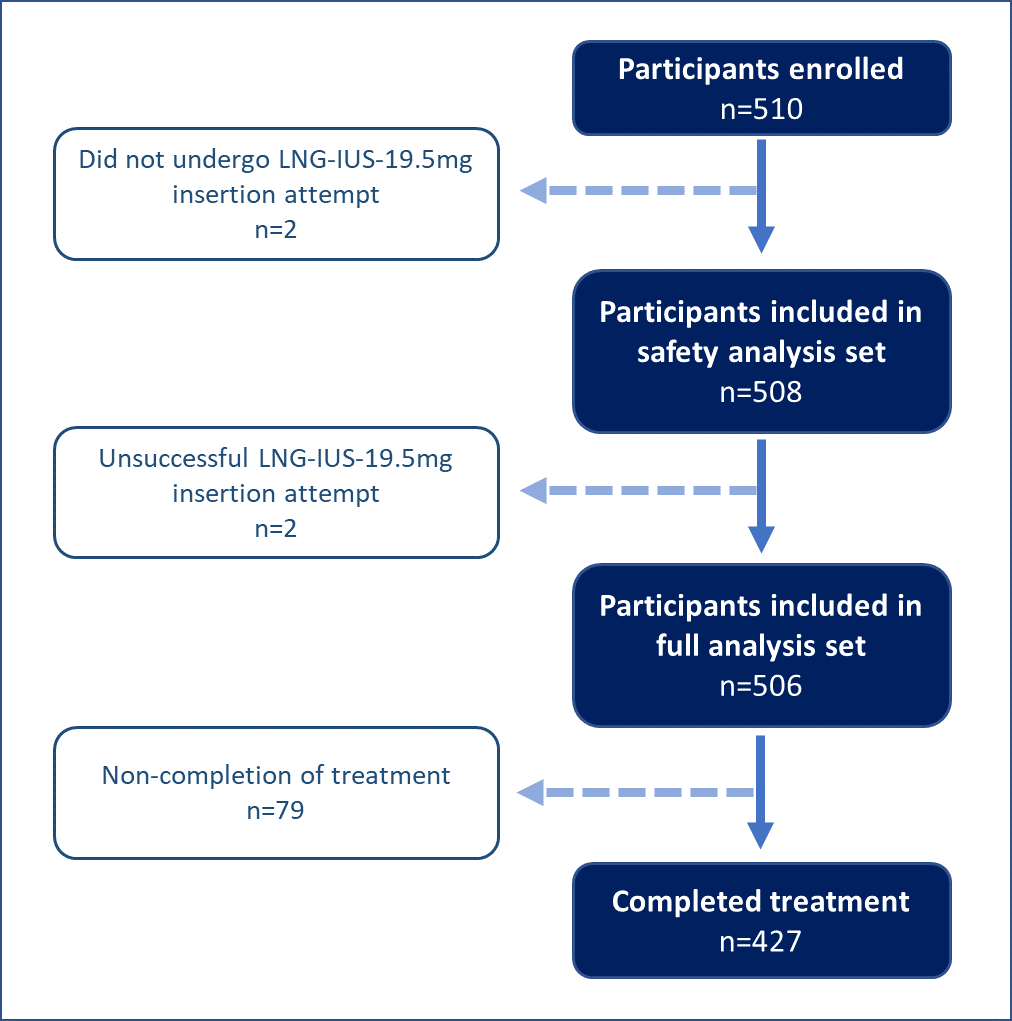


KYSS: Kyleena^TM^ Satisfaction Study.

**Fig. S2** Prior method of contraception in the German population (safety analysis set)

IUD: intrauterine device; IUS: intrauterine system.

**Fig. S3** Motivation for choosing LNG-IUS-19.5mg in the German population (safety analysis set)^a^

^a^More than one reason could be given.

LNG-IUS: levonorgestrel-releasing intrauterine system.

**Fig. S4** Ease of placement (**a**), pain during placement (**b**), and additional measures used during placement (**c**) of LNG-IUS-19.5mg in the German population (safety analysis set), stratified by parity and age

LNG-IUS: levonorgestrel-releasing intrauterine system.
